# Barium Appendicitis 6 Weeks After Upper Gastrointestinal Imaging

**DOI:** 10.3389/fped.2020.00535

**Published:** 2020-09-02

**Authors:** Gang Shen, Kang Sun, Zhe Fan

**Affiliations:** ^1^Department of Pediatric Surgery, Dalian Children's Hospital of Dalian Medical University, Dalian, China; ^2^Department of Digestive Endoscopy, The First Affiliated Hospital of Dalian Medical University, Dalian, China; ^3^Department of General Surgery, The Third People's Hospital of Dalian, Dalian Medical University, Dalian, China

**Keywords:** barium appendicitis, barium sulfate, upper gastrointestinal imaging, complication, child

## Abstract

Barium sulfate is widely used for gastroenterology imaging. Retention of barium in the appendix, where it acts as an appendicolith, thereby leading to obstruction and inflammation. Barium-associated appendicitis is a very rare complication of upper gastrointestinal imaging (UGI), especially in children. We present a case involving an 8-year-old girl who was diagnosed with acute appendicitis due to a barolith and required a laparoscopic appendectomy 6 weeks after UGI. After UGI, patients should be informed regarding possible retention of barium in the appendix, which can cause acute appendicitis. Then, a laparoscopic appendectomy was performed successfully. We should be cautious of this potential risk to prevent complications with early intervention in children.

## Background

Acute appendicitis of children is one of the most common surgical problems encountered by pediatric surgeons. An obstruction of the appendiceal lumen can result in the development of acute appendicitis (1, 2). Upper gastrointestinal imaging (UGI) follow-through is a radiologic investigation to delineate the stomach and intestinal anatomy. Barium sulfate is inert and not harmful to the mucosa, and is widely used and is considered safe in gastrointestinal imaging studies (3). Barium sulfate is not harmful in gastrointestinal imaging studies in children suspected to have a gastrointestinal tract malformation, chronic constipation, and unexplained abdominal pain. Barium-related appendicitis of children is a very rare complication of barium studies for imaging the gastrointestinal system. Here, we present a patient with acute appendicitis associated with barium retention in the appendix.

## Case Presentation

An 8-year-old girl was admitted to our clinic complaining of abdominal pain for 3 days. She denied any other gastrointestinal or genitourinary symptoms. The abdominal pain originally was periumbilical in nature, but later spread to the right lower quadrant. She had undergone an UGI study of her stomach 6 weeks earlier as a routine evaluation for gastric volvulus.

**Figure 1:**
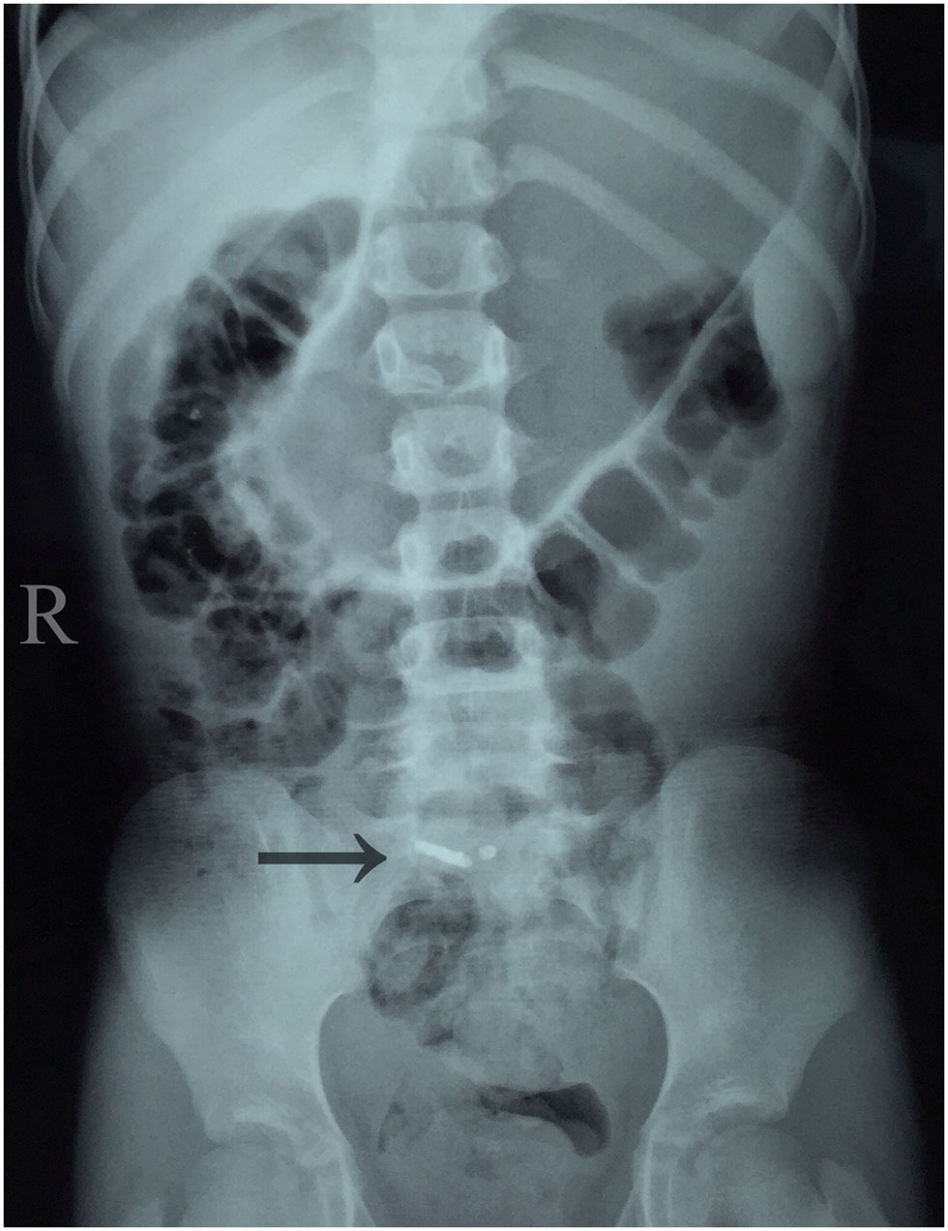
Abdominal plain film showing radiopaque tubular structure around the ileocecal junction in the right lower quadrant.

**Figure 2:**
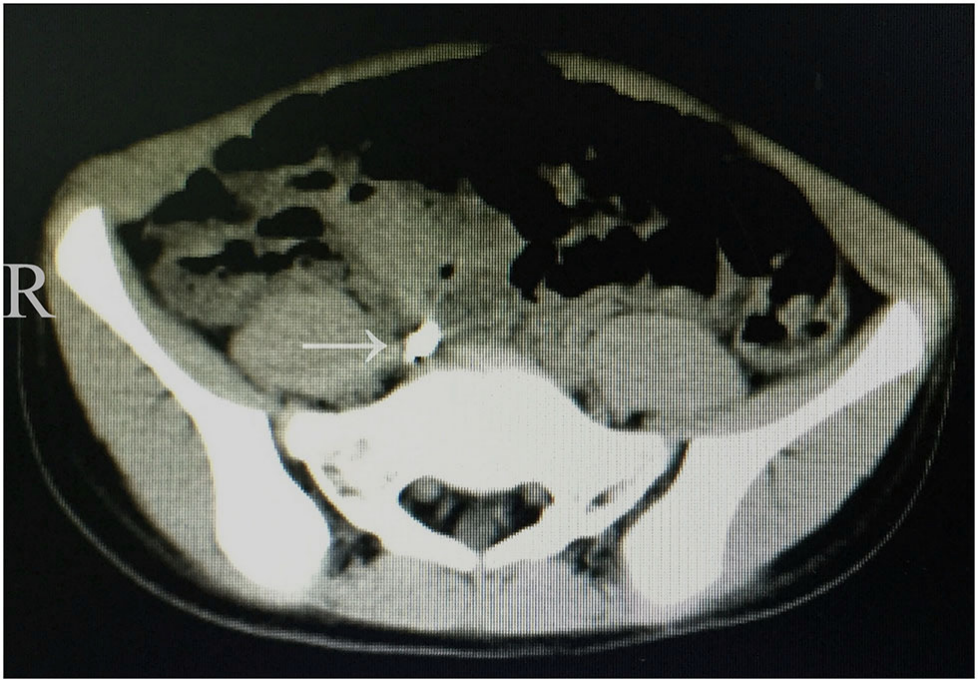
Axial CT scan illustrating high-density material adjacent to the cecum and medial to the psoas major.

On physical examination, the girl had a temperature of 37.3°C and had moderate abdominal pain. Her abdominal examination revealed discomfort over McBurney's point and guarding in the right lower quadrant, although no peritoneal signs were present. Blood testing revealed that the white blood cell count was 13 × 10^9^/L with 78% neutrophils and a C-reactive protein concentration of 30 mg/L. Other laboratory study results were normal. A plain abdominal radiograph revealed a radiopaque tubular structure in the right lower quadrant (Figure 1). A computed tomography (CT) scan showed high-density material near the cecum (Figure 2). Multiplanar reconstruction of the CT images demonstrated the appendix filled with high-density material. Therefore, acute appendicitis was highly suspected.

The patient underwent an urgent laparoscopic appendectomy. The resected appendix was shown to be inflated and contained barium. A pathologic examination of the operative specimen showed typical findings of acute appendicitis. A diagnosis of barium appendicitis was therefore established. The patient made a rapid recovery and was discharged on the 4th post-operative day. At the 5-month follow-up evaluation, there were no post-operative complications; long-term follow-up evaluations are planned.

## Discussion

Because barium sulfate crystals are not absorbed by the mucosa and are not soluble in gastrointestinal fluid (4), barium is not harmful to the gastrointestinal mucosa and widely used for UGI. As an alternative selection to barium, gastrografin has been used for UGI and gastrografin produces higher delineation for esophagus compared with barium sulfate (5). Barium appendicitis is a rare complication of barium swallow in children. Some authors have suggested that barium studies are associated with an increased, time-dependent appendicitis risk (6). Barium appendicitis is defined as follows: (1) a history of a prior barium examination; (2) diagnosed with acute appendicitis; and (3) CT scan shows a high-density substance in the appendiceal lumen. Patients who meet all three conditions are deemed to have barium appendicitis (2).

The mechanism of the onset of barium appendicitis is the result of obstruction of the appendiceal lumen by barium sulfate, as well as ordinary fecaliths; The appendix is filled with nearly all materials used during a barium examination; however, most patients will have spontaneous evacuation of the barium from the appendix within 48 h, although in children this may take more time (7). In our case, the barium was retained in the appendiceal lumen and acute appendicitis developed 5 weeks after UGI.

Barium appendix can be confused with metal foreign bodies and in the present case (8), the possibility of foreign bodies existed because swallowing of foreign bodies is well-known phenomenon among children. Therefore, a history of a UGI was the main clue ruling out foreign bodies.

An appendectomy should be performed if a clinical suspicion of acute appendicitis or chronic right lower quadrant stress exists with retained barium in the appendiceal lumen, to prevent the risk of development of acute appendicitis with perforations (8, 9). In our case, the pain was originally periumbilical, but later spread to the right lower quadrant. The laboratory parameters were abnormal, and the physical examination and CT scan findings were suggestive of acute appendicitis. A laparoscopic appendectomy was performed.

## Conclusion

Barium appendicitis is a rare clinical entity in children, which should be distinguished from foreign bodies. After barium studies, patients should be informed regarding possible retention of barium in the appendix, which can cause acute appendicitis. Thus, if abdominal pain develops, we should be cautious of this potential risk to avoid delayed diagnosis and appropriate treatment; and a laparoscopic appendectomy could be a good choice.

## Data Availability Statement

The raw data supporting the conclusions of this article will be made available by the authors, without undue reservation.

## Ethics Statement

The studies involving human participants were reviewed and approved by Ethics Committee of Dalian Children's Hospital of Dalian Medical University. Written informed consent to participate in this study was provided by the participants' legal guardian/next of kin. Written informed consent was obtained from the individual(s), and minor(s)' legal guardian/next of kin, for the publication of any potentially identifiable images or data included in this article.

## Author Contributions

GS and KS: conceptualization and data curation. ZF: investigation, validation, and writing of the original draft. All authors contributed to the article and approved the submitted version.

## Conflict of Interest

The authors declare that the research was conducted in the absence of any commercial or financial relationships that could be construed as a potential conflict of interest.
